# ColorX: A Fitness Tracker-Based Device for Rapid, Optical Sensing of Water Quality Parameters

**DOI:** 10.3390/s25164935

**Published:** 2025-08-09

**Authors:** Venkata V. B. Yallapragada, Adarsh Ananthachar, U. Gowda, F. ní Chlochasaigh, L. O’Faolain, G. C. R. Devarapu

**Affiliations:** 1 Center for Advanced Photonics and Process Analysis, Munster Technological University, T12P928 Cork, Ireland; 2 Tyndall National Institute, T12R5CP Cork, Ireland

**Keywords:** colorimetric sensor, fitness tracker, nitrite, sulphate, chromium, free-chlorine, turbidity

## Abstract

Optical sensors have emerged as a popular technology for sensing biological and chemical analytes in various fields, including environmental monitoring, toxicology, disease/infection screening, and food processing, due to their ease of use, high sensitivity, and specificity. In this study, we introduce ColorX, an ultra-portable and smart spectrophotometric device based on a commercially available fitness tracker. ColorX exploits the in-built LEDs and photodiodes of a fitness tracker for wavelength-specific absorption measurements and can be controlled wirelessly using a companion smartphone app. The device’s raw data are transmitted via Bluetooth and stored on the app for analysis and data visualisation. We validated the performance of ColorX against a standard benchtop spectrophotometer by experimentally testing five different measurements related to water quality: nitrite (>0.07 mg/L, %avgCV: 1.06)), sulphate (>18 mg/L, %avgCV: 0.39), chromium (>0.002 mg/L, %avgCV: 0.51), free chlorine (>0.005 mg/L, %avgCV: 0.68), and turbidity (>2.97 NTU, %avgCV: 1.04). Our results showed that ColorX had comparable performance to the benchmark spectrophotometer (R^2^ values > 0.9 in all cases). Due to its ultra-portability, water-proof design, wireless control, and smartphone-aided data analysis, we believe ColorX will be highly beneficial for a wide range of on-field spectrophotometric applications. Our work demonstrates the potential of frugal science to develop affordable and accessible technology for optical sensing.

## 1. Introduction

Monitoring the quality and quantity of a substance is a critical task in various fields, such as measuring pollutants in environmental monitoring, testing for hazardous substances in toxicology, quality testing in food and beverage industries, etc. [1,2,3]. Traditional methods of measuring and analysing organic and inorganic analytes involve sophisticated, large instruments that require expert supervision and are confined to laboratory settings. The confinement of these analyses to laboratory-based settings has many disadvantages that arise from the time and risk involved in the transportation of samples to the facility and need for a dedicated technician. The instruments used in traditional laboratories are bulky and not suitable for on-field and real-time sensing demands. However, recent advances in 3D printing [4,5], low-cost electronics, miniaturisation of technology, and open-source initiatives like FOSS (free and open-source software) [6,7] have helped in the development of low-cost sensors with high on-field ease of deployability.

**Figure 1:**
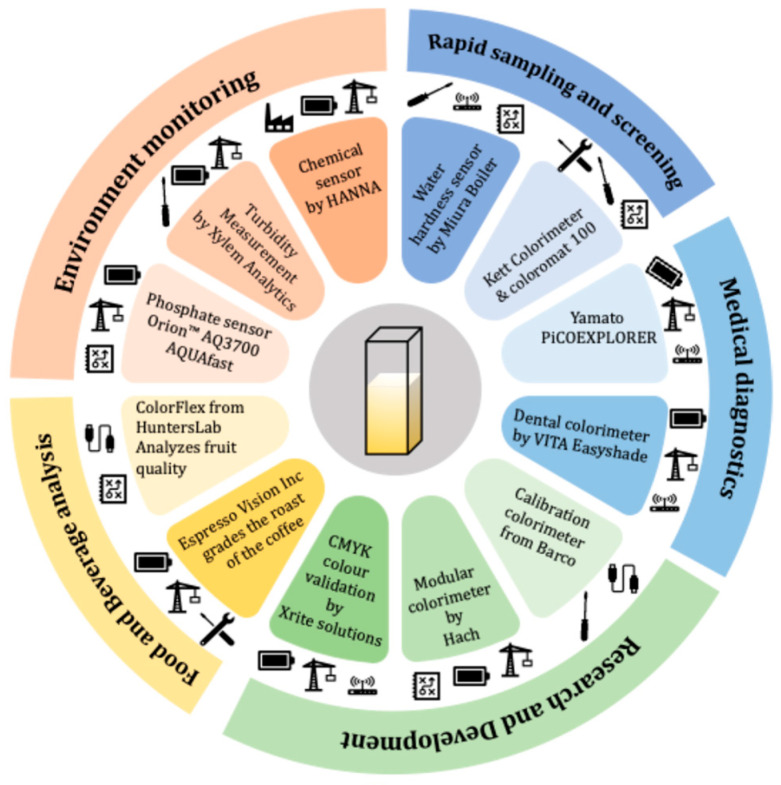
Various applications of colorimetric sensors. Commercial applications of colorimetry are wide, and a broad range of the devices is currently present in the consumer market. Different applications are highlighted with available devices in the consumer market and their respective features [31,32,33,34,35,36,37,38,39,40,41,42,43,44,45,46].

In recent years, there has been tremendous growth in portable biochemical sensing technologies with life science application platforms [8] and quality control devices [9] dominating the commercial handheld sensor space. For many of these applications, devices measuring various optical properties such as fluorescence [10], optical density [11], and colour change [12] seem attractive for their non-invasive nature and simple hardware implementation. The need for rapid and real-time biochemical sensing has also been greatly emphasised during the COVID-19 pandemic [13]. The development of rapid sensing technology for detecting spike protein of the SARS nCoV2 viral capsid using antibodies and loop-mediated isothermal amplification (LAMP)-based assays for Covid detection has been achieved by leveraging low-cost optical sensors. These sensors can be manufactured in large numbers in relatively short periods, making them ideal for quick on-field deployment. Amongst many of these optical sensing technologies, colorimetric sensing has several advantages such as the ability to be detected by the naked eye, quick result turnaround time, high sensitivity, and relative low-cost of hardware components [14]. Colorimetry works on the principle of change in the intensity of the electromagnetic spectrum at a specific wavelength in the visible region due to the presence and/or amount of an analyte [15]. Traditionally, colorimetry was performed by visual inspection [16]. For example, qualitative colorimetric applications such as pregnancy testing kits and urine dipsticks and semi-quantitative tests such as litmus tests for solvents can be detected by human eyes, without the need for detection hardware by examining for a distinctive colour change. However, for quantitative methods, accurate measurements are required to estimate the exact amount of an analyte present in a solution. In such cases, camera-guided computational analysis [17,18,19,20,21,22,23,24] or spectroscopy [25,26,27,28,29,30] are used as a detection method. Early versions of such colorimetric devices are bulky and limited to laboratory settings. Such devices are still a standard tool in research and industrial settings. Figure 1 provides an overview of various colorimetric sensor applications including commercially available devices for each respective application.

To deploy colorimetric sensors for in-field applications, simple and portable devices are required. Smartphone based camera-guided colorimetric sensors offer an affordable and a semi-automated solution. The availability of an app-based control interface and analysis platform also adds to the better user experience. However, smartphone-based colorimetric devices are best suited for one-time measurement and are not practical for high throughput and continuous measurements due to the use of power-hungry hardware components (i.e., cameras and display screens) in a smartphone. Thus, these devices are not suitable for high-throughput, off-grid, and remote monitoring settings.

**Figure 2:**
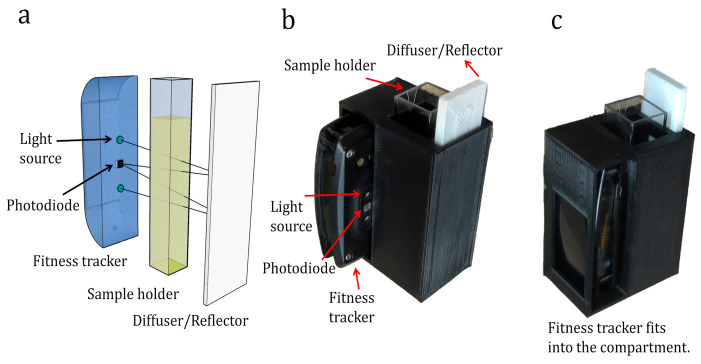
(**a**) Working principle of reflectance-based colorimetry. The light being emitted by the in-built LEDs of the fitness tracker passes through the sample holder. A reflector, placed at the end of the sample holder, reflects back the light. The reflected light passes back again through the sample holder and reaches the in-built photodiodes of the fitness tracker. The amount of light detected by the photodiode depends on the concentration of the analyte in the sample solution. (**b**) Fitness tracker, sample holder, and diffuser placed along with a 3D-printed enclosure. (**c**) Front view of the ColorX after complete assembly.

The opto-electronic devices present in modern wrist-worn fitness trackers [47,48] have had a huge impact through the provision of crucial information on body vitals as well as health and wellness metrics by monitoring the physiological signs of users. Most of the non-invasive optical sensors in a fitness tracker/smartwatch device are in essence colorimetric sensors as they monitor changes in skin colour. Each cardiac cycle of the heart results in blood volume changes in the microvascular bed of skin tissue. Therefore, by monitoring the skin colour change, it is possible to measure vital signs such as heart rate, SpO2, and blood pressure [32]. Among various optoelectronic sensors present in the smart wearables, the heart rate sensor is the most commonly found component even in low-cost fitness tracker devices. A typical fitness tracker’s heart rate sensor includes LEDs for illumination, photodiodes for detection, an OLED screen, and a Li-ion battery to power the system. Advancements in solid-state lighting have facilitated the miniaturization of these sensors [49,50,51]. Particularly, the development of LEDs, which are quasi-monochromatic and highly efficient [52], made a huge difference. Commercial fitness trackers pack these optical sensors with LEDs and photodiodes in a compact arrangement that can be adapted for colorimetric detection of various biochemicals. In a previous study [11], we demonstrated continuous optical density monitoring using a fitness tracker-based device called ODX. Inspired by the capabilities of fitness trackers and with the goal of increasing the versatility of biochemical sensing, we have developed ColorX, an ultraportable smart colorimetric device that utilizes the compact opto-electronic hardware of a generic fitness tracker. In contrast to standard benchtop devices, ColorX operates on the transreflectance principle [53]. As illustrated in Figure 2a, the LED emits light with a wavelength that corresponds to the absorption band of a sample. After interacting with the sample, a fraction of light is transreflected from the diffuser at the back of the sample holder and detected by the photodiode, depending on the concentration of the sample.

This transreflectance-based configuration enables the design of colorimetric sensors in fitness trackers where the light source and the photodetector can be located on the same side, resulting in simplified hardware with a single optical window. The use of a single optical window is highly desirable for the miniaturisation and liquid leak-proofing colorimetric optical sensors in fitness trackers. We exploited these transreflectance-based optical sensors of a fitness tracker in ColorX for colorimetric detection of analytes in water. As schematically shown in Figure 2a, the incident light in the ColorX device undergoes multiple reflections within the sample holder. This enables a long effective path length on a relatively small sensor footprint, as demonstrated by the matchbox-sized device in Figure 2b,c. Thanks to the innovative application of optical sensors from a fitness tracker in ColorX, the only components needed to build the ColorX are a 3D-printed enclosure to hold the sample cuvette and a 3D-printed diffuser to enable the transreflectance. With its portable handheld form, ColorX can be deployed anywhere and controlled wirelessly through its in-built Bluetooth module. The ColorX device has been precisely calibrated, and its functionality has been compared to a standard benchtop spectrophotometer. To validate ColorX, it was used to measure five different parameters related to water quality: nitrite, sulphate, free chlorine, chromium, and turbidity. In all cases, ColorX’s performance was on par with the benchtop instrument. Additionally, a smartphone companion app has been developed to communicate with the ColorX device. This app acts as an interface for data visualisation and also to share the data across various platforms. This article discusses the design development and testing of ColorX.

## 2. Materials and Methods

### 2.1. ColorX Hardware Parts and Components

ColorX hardware consists of the following: 1. a fitness tracker, 2. 3D-printed enclosure, and 3. a diffuser.

**Figure 3:**
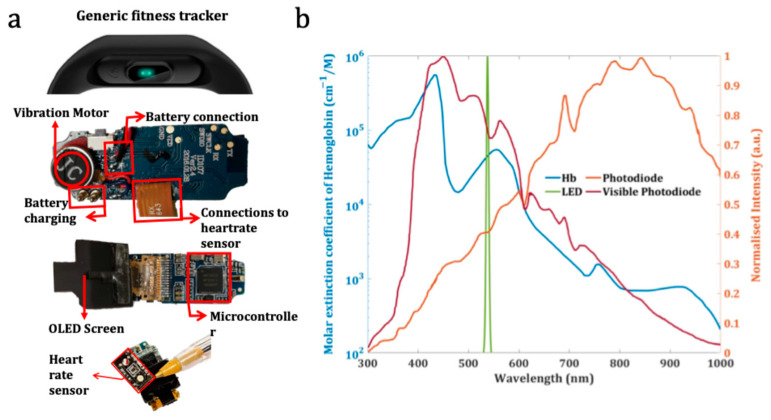
(**a**) A generic fitness tracker. The back face is shown with in-built LEDs and other crucial internal electronic components. (**b**) Emission spectra of the in-built LED, the detection range of the photodiodes along with the absorption curve of haemoglobin. The green LEDs are used to detect oxygenated haemoglobin in blood and can be leveraged for other sensing applications.

**Fitness tracker:** The chosen fitness tracker for the ColorX is an ID107HR branded device from Shenzhen DO Intelligent Technology Ltd., which costs between USD 10 and 25 (See Figure 3a). This same fitness tracker was used in our previous work [11]. This ID107HR fitness tracker consists of an nRF5122 (Nordic Semiconductor Inc., Trondheim, Norway) microcontroller that can be programmed using open-source firmware development tools based on an Arduino IDE [54]. The heart rate sensor in this fitness tracker uses the Si1143 IC, Silion Labs, Austin, Texas [55], which employs two photodiodes that cover both the infrared (IR) and visible spectral ranges. In Figure 3b, we show the broadband response of these visible and IR photodiodes with an orange line and a red line, respectively. The heart rate sensor also has two identical green LEDs positioned on either side of the Si1143 IC. The emission spectrum of these LEDs is shown with a green solid line in Figure 3b. In the ID107HR fitness tracker used in this study, Si1143 IC drives the LEDs and reads the raw data from the photodiodes. This fitness tracker has an in-built battery of 70 mAh, and a single charge enabled 150–200 test cycles before recharge was needed.

**Figure 4:**
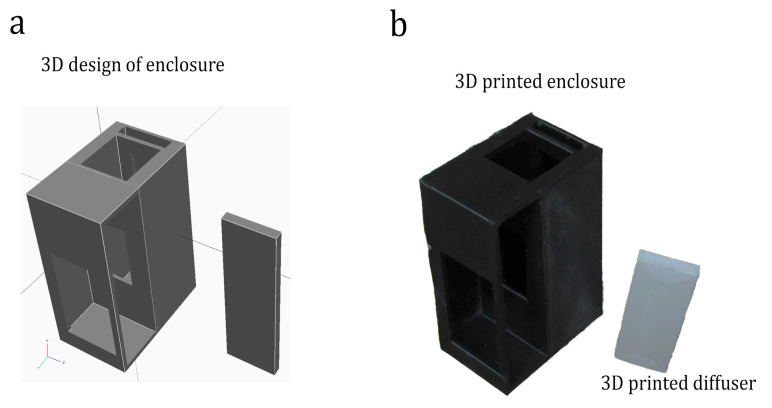
(**a**) Three-dimensional design for the enclosure. The design was created using openSCAD free to use design tools. (**b**) Three-dimensional-printed part: The model was 3D printed using Ultimaker 3 and PLA filament.

**3D-printed enclosure:** A 3D-printed enclosure (see Figure 4) has been developed to house the fitness tracker and the sample holding space for a standard cuvette and a diffuser. Open-source parametric CAD software (OpenSCAD version 2015.03-2) was used to design the enclosure, which was then fabricated using an Ultimaker 3D printer. To eliminate external light influence and reflections, black polylactic acid (tough PLA) material was used for the enclosure, and white PLA material was used for the diffuser to reflect back as much light as possible. The corresponding .STL files were sliced using CURA (Version 3.2.1) to generate the G-CODE required for the 3D printing process. The printing parameters used were as follows: 0.4 mm nozzle, 0.1 mm layer height, and 20% infill without any adhesion and support structures. Complete design files for Colorx, including 3D models, firmware and mobile application are made available as open source under GPL 3.0 licence at https://github.com/V77VV/ColorX (accessed on 1 May 2024).

**Figure 5:**
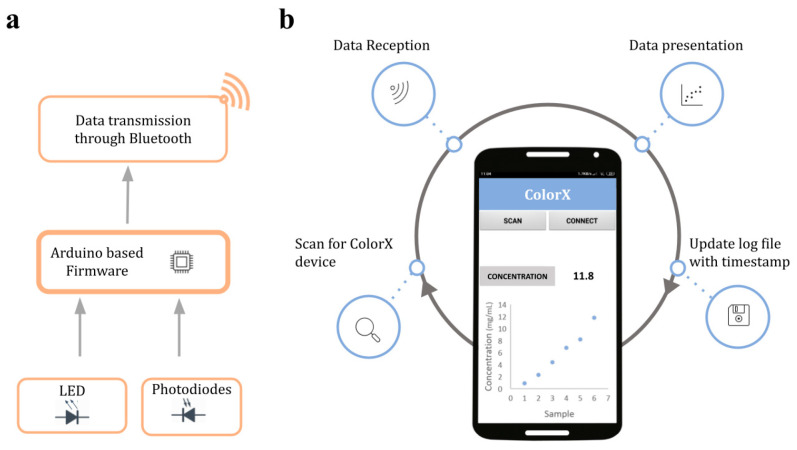
Functional workflow of ColorX device. (**a**) ColorX firmware workflow. (**b**) ColorX app features.

**Firmware:** The processor for ColorX is a nRF51822a microcontroller in the fitness tracker. It was programmed using the Arduino integrated development environment (IDE) and an Arduino compatible toolset for nRF5 series microcontrollers developed by Sandeep Mistry [56]. To access peripheral components such as LEDs, photodiodes, the Bluetooth low energy module, and the OLED display screen of the fitness tracker, additional libraries were installed in Arduino IDE. These libraries are essential to integrate and establish communication between the microcontroller and enable data sharing to the smartphone using Bluetooth. The workflow of the firmware is schematically shown in Figure 5a. The custom firmware developed for the ColorX is uploaded to the microcontroller through the soldering pads GND, VCC, SCL, and SDIO on the fitness tracker’s circuit board. A Black Magic Probe Mini V2.1 (1BitSquared Ltd., Eugene, OR, USA.) program was utilized for this purpose.

Smartphone App Development: ColorX app was developed using MIT App Inventor, an open-source Android app development platform [57]. The app has a simple user-friendly interface to enable data transfer from the ColorX device to the smartphone wirelessly via Bluetooth. The main function of the app is to process raw data and display it on the smartphone screen. The app also saves the data in a .txt format inside the smartphone’s internal storage which can be transferred to any device. The graphical interface and functioning of the app are shown in Figure 5b.

### 2.2. Standard Solutions for ColorX Testing

**Nitrite detection:** Griess reagent kit (ThermoFisher Cat No: G7921) was used to test nitrite in water. Briefly, 1 mM nitrite standard solution was diluted to 10 mL samples in the range of 1–100 uM. Then, 300 uL of nitrite sample was added to 2.6 mL of distilled water in a cuvette. Next, 100 uL of Griess reagent (50 uL N-(1-naphthyl) ethylenediamine dihydrochloride + 50 uL sulphanilic acid) was then added to each cuvette. All sample mixtures were incubated at room temperature for 30 min, and absorbance at a wavelength of 536 nm was recorded using a standard benchtop spectrophotometer (Shimatsu UV1280). The same samples were also tested using ColorX and the values from the VIS and IR photodiodes were recorded using the ColorX app.

**Sulphate detection:** SulfaVer 4 kit (Hach Cat No: 2106769) was used for testing sulphate in water. A sulphate standard solution of 2500 mg/L (Hach Cat No: 1425249) was used to make 10 mL dilutions in the range of 0.1–70 mg/L. Here, 100 mg of SulfaVer 4 reagent was added, and the samples were incubated for 20 min at room temperature. Absorbance at a wavelength of 536 nm was recorded using a standard benchtop spectrophotometer (Shimatsu UV1280). Same samples were also tested using ColorX, and the values from the VIS and IR photodiodes were recorded using the ColorX app.

**Free chlorine detection:** Free chlorine in water samples was detected using Hach DPD method, which uses powdered pillows of DPD reagent (Hach Cat No: 2105569). The detection range of the kit used is 0.02–2.00 mg/L. Different standard concentrations (0.01–2 mg/L) were made by diluting the stock solution of free chlorine (Hach Cat No: 1426810). Absorbance at a wavelength of 540 nm was recorded using a standard benchtop spectrophotometer (Shimatsu UV1280). Same samples were also tested using ColorX and the values from the VIS and IR photodiodes were recorded using the ColorX app.

**Chromium detection:** ChromaVer 4 reagent was used to detect Chromium in water. Chromium standards (Hach Cat No: 81042H) were purchased and diluted with Millipore water as per the manufacturer’s instructions (dilution range 0.01–0.08 mg/L). Powdered pillows of ChromaVer 4 reagent (Hach Cat No: 1271099) were used according to the manufacturer protocol to detect different standard concentrations of chromium in water samples. Detection range of the kit used is up to 0.70 mg/L. Absorbance at a wavelength of 540 nm was recorded using a standard benchtop spectrophotometer (Shimatsu UV1280). Same samples were also tested using ColorX, and the values from the VIS and IR photodiodes were recorded using the ColorX app.

**Turbidity:** Turbidity measurements were performed using a formazine solution as the turbidity standard. The formazine solution, with a stock concentration of 4000 NTU was obtained from Hach (Cat No: 246149). To obtain different optical density (OD) standards, different OD standards (range: 0.1–1 OD) were made by diluting the standard solution with Millipore water. Absorbance was read at a wavelength of 600 nm using a standard benchtop spectrophotometer (Shimatsu UV1280). Same samples were also tested using ColorX, and the values from the VIS and IR photodiodes were recorded using the ColorX app.

Note that all the above standard solutions are generated and the corresponding colorimetric experiments are conducted in triplicate.

## 3. Results and Discussion

**Figure 6:**
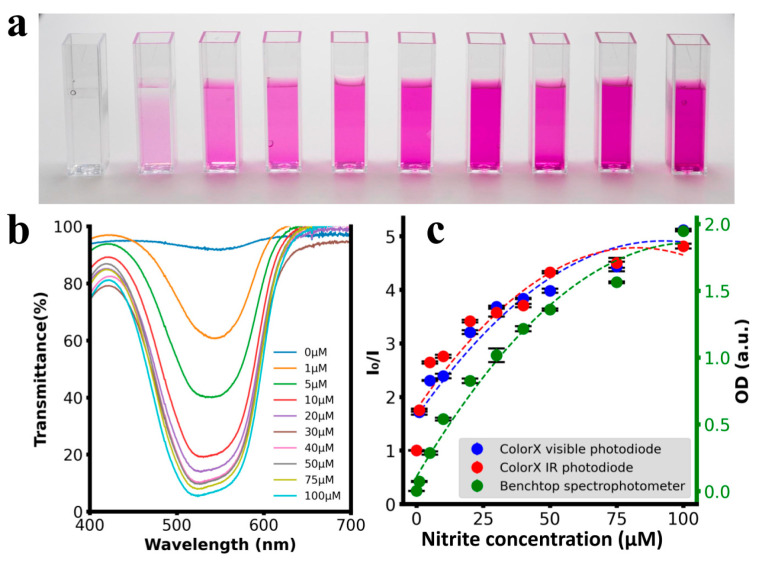
Detecting nitrite using ColorX. (**a**). Photograph of colorimetric reactions for nitrite in cuvettes (**b**). Absorbance spectra of different samples (1–100 uM) are shown. (**c**). Absorbance values from the visible and IR photodiodes on the ColorX device along with the absorbance values from the benchtop spectrophotometer were plotted. All the samples corresponding to each test and their absorbance spectra (400–700 nm) are also shown.

**Figure 7:**
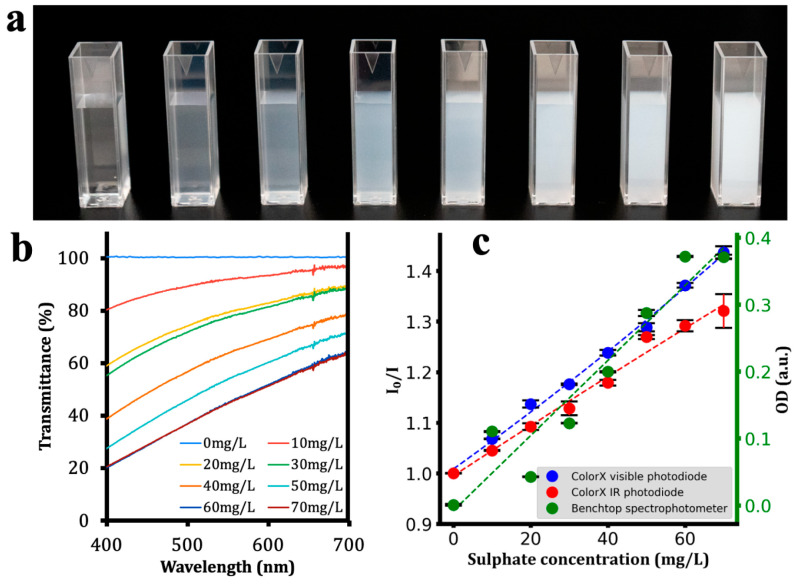
Detecting sulphate using ColorX. (**a**). Photograph of colorimetric reactions for sulphate in cuvettes (**b**). Absorbance spectra of different samples (0–70 mg/L) are shown. (**c**). Absorbance values from the visible and IR photodiodes on the ColorX device along with the absorbance values from the benchtop spectrophotometer were plotted. All the samples corresponding to each test and their absorbance spectra (400–700 nm) are also shown.

**Figure 8:**
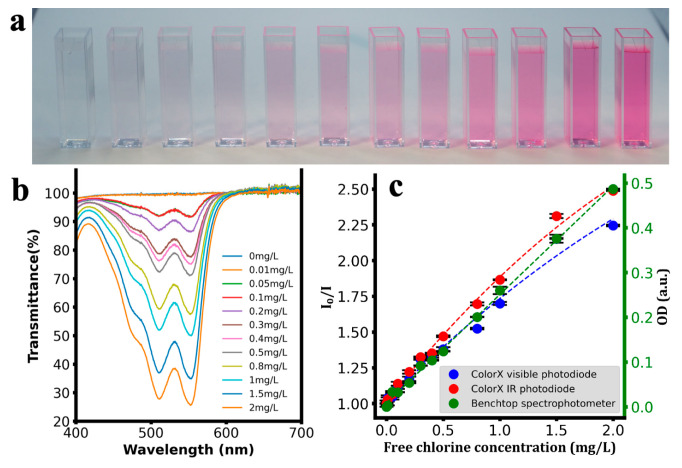
Detecting free chlorine using ColorX. (**a**). Photograph of colorimetric reactions for free chlorine in cuvettes (**b**). Absorbance spectra of different samples (0–2 mg/L) are shown. (**c**). Absorbance values from the visible and IR photodiodes on the ColorX device along with the absorbance values from the benchtop spectrophotometer were plotted. All the samples corresponding to each test and their absorbance spectra (400–700 nm) are also shown.

**Figure 9:**
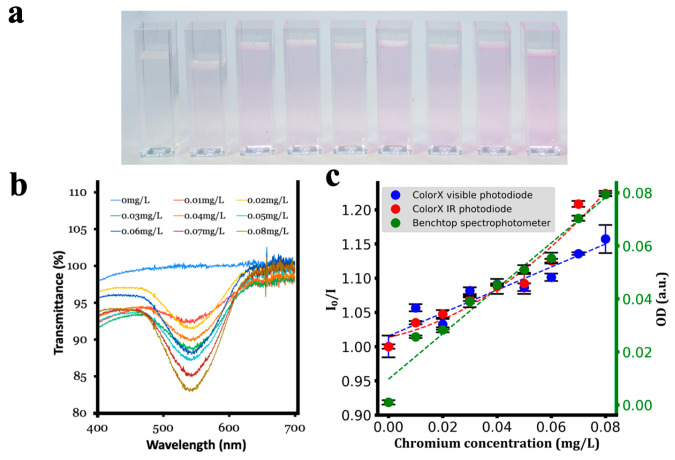
Detecting chromium using ColorX. (**a**). Photograph of colorimetric reactions for chromium in cuvettes (**b**). Absorbance spectra of different samples (0–0.08 mg/L) are shown. (**c**). Absorbance values from the visible and IR photodiodes on the ColorX device along with the absorbance values from the benchtop spectrophotometer were plotted. All the samples corresponding to each test and their absorbance spectra (400–700 nm) are also shown.

**Figure 10:**
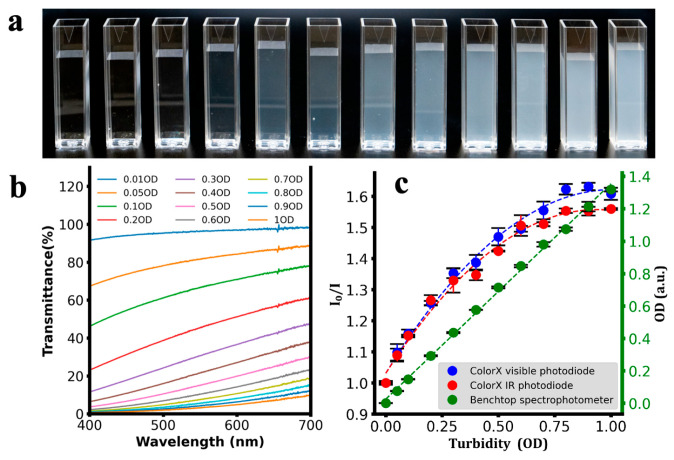
Detecting turbidity using ColorX. (**a**). Photograph different levels of turbidity in cuvettes (**b**). Absorbance spectra of different samples (0–1.0 OD) are shown. (**c**). Absorbance values from the visible and IR photodiodes on the ColorX device along with the absorbance values from the benchtop spectrophotometer were plotted. All the samples corresponding to each test and their absorbance spectra (400–700 nm) are also shown.

After assembling the ColorX device, its performance was evaluated by conducting colorimetric assays to detect nitrite, sulphate, free chlorine, chromium, and turbidity as shown in Figure 6, Figure 7, Figure 8, Figure 9 and Figure 10. For each analyte, at least eight different concentrations were used in triplicate. The images of the colour changing reaction cuvettes and their corresponding spectra obtained with the standard benchtop spectrophotometer are shown in (a) and (b) of Figure 6, Figure 7, Figure 8, Figure 9 and Figure 10 respectively.

### 3.1. ColorX vs. Benchtop Spectrophotometer

These colorimetric reaction cuvettes were tested in parallel using both the ColorX device and a standard benchtop spectrophotometer (Shimatsu UV1280). The data obtained from the benchtop spectrometer were used to establish a correlation between the raw values of the VIS and IR photodiodes in the fitness tracker and the concentration values of the analytes. These validation results are presented in the subplots corresponding to (c) of Figure 6, Figure 7, Figure 8, Figure 9 and Figure 10. The absorption values of the analytes from the benchtop spectrophotometer (green circles) and from the IR (blue circles) and VIS (red circles) normalised photodiodes (I/I0) of the fitness tracker are collected and plotted against the concentration values, where I0 represents the photodiode values of the blank sample, which is deionised (DI) water. These circles on the plots represent the average value of the triplicate samples. Trend lines are fitted through these circles to obtain the regression equations along with the corresponding R^2^ values, which are shown in Table 1. It is clearly evident from these plots and the R^2^ values in Table 1 that ColorX performed with an accuracy similar to a standard benchtop spectrophotometer. Coefficients of variation (CVs) for triplicate measurements at each concentration level were calculated to access the precision of ColorX device. The CV was determined using CV(%) = (σ/μ) × 100, where σ is the standard deviation, and μ is the mean of three replicates. The average CV across all concentrations (%avgCV) of less than 2% represents excellent overall measurement precision of a device. As show in Table 2, the %avgCV for ColorX device ranged from 0.39 to 1.21 for the visible photodiode and from 0.51 to 1.10 for the infrared photodiode across all tested analytes, demonstrating excellent measurement precision.

The limit of detection was determined according to the criterion outlined by International Conference on Harmonisation (ICH) [58]. According to the ICH, LOD is determined by 3.3 σ/m, where σ is the standard deviation of blank values predicted by calibration equations, and m is the slope of the calibration plot in the linear range of the calibration curve. Using this method, we calculated LODs for all analytes and compared them to regulatory standards. Table 2 shows EPA/CDC recommended detection limits for nitrite, sulphate, free chlorine, and chromium in drinking water. Our experiments with ColorX demonstrated that the device was able to measure concentrations approximately matching these guidelines. Furthermore, ColorX was also capable of measuring turbidity, which is a widely used indicator of water quality. Multiple factors such as microbial contamination as well as the presence of chemicals and organic wastes influence the colour and turbidity of water. ColorX detected turbidity at standards that are significantly below the detection limits of a naked eye [59]. While the lowest turbidity levels are slightly above the EPA limits, it is important to note that 2.97 NTU is an extremely low level of turbidity and is very difficult to measure even with standard benchtop spectrophotometers. Note that both VIS and IR photodiodes have comparable accuracy based on their R^2^ values (Table 1) and LOD values (Table 2).

The fitness tracker used in the ColorX device has two in-built photodiodes, namely VIS and IR. In all the experiments, both the photodiode responses have been recorded. It should be noted that the calibration curves for these photodiodes (shown on the left Y-axis of the graphs) were non-linear, in contrast to the linear calibration curves obtained from the spectrophotometer (shown on the right Y-axis). The non-linearity in ColorX’s calibration is primarily a consequence of the transreflectance principle, as explained earlier, where multiple internal reflections occur between the light source and detector. Additional factors contributing to non-linearity include spectral mismatch between LED emission and reagent absorption bands, LED spectral width, broadband photodiode wavelength sensitivity, and stray light contributions arising from incomplete light shielding of enclosure. In this study, the performance of ColorX was validated experimentally using five different analytes as a proof-of-concept, including nitrite, sulphate, chromium, free chlorine and turbidity. These analytes were chosen as they are key parameters analysed in various domestic as well as industrial activities, such as water quality monitoring for agriculture [60], drinking water supplies [61], and wastewater treatment plants [62].

### 3.2. Advantages of ColorX vs. Existing Colorimetric Solutions

Although we demonstrated ColorX’s capability to detect five analytes, it has the potential to detect a variety of other analytes depending on the availability of colorimetric reagents with absorption bands that match the emission spectra of the green LEDs in the heart rate sensors, such as glucose [63] and calcium [64]. Additionally, LEDs in fitness trackers can be changed or extra LEDs can be added [11] to match the colour change of the reagents. It is worth noting that many fitness trackers now come equipped with multiple colour LEDs to measure various physiological parameters [65,66,67]. In addition, it is interesting that many fitness trackers are available with open-source hardware and software designs, so that they can be modified potentially as ColorX devices. When compared to a benchtop instrument, ColorX has significant advantages such as continuous data collection, wireless control, and a smartphone-based companion app to store, analyse, and share data across different platforms. Furthermore, we made the design files of the 3D-printed enclosure, diffuser, firmware, Android app, and build process of ColorX completely open source under the GPL 3.0 license. This allows ColorX to be adopted for other colorimetric applications. Using a generic fitness tracker makes ColorX a pocket-sized ultraportable system. Once the custom firmware for ColorX is installed on a fitness tracker, there is no need for human calibration of the device. All experiments can be remotely monitored on the smartphone app via Bluetooth.

ColorX is also an excellent example of a frugal science invention that has a potential for deployability outside the laboratory. ColorX is designed to be low-cost and affordable, making it accessible to a larger population. Low cost becomes a significant factor for deploying science in regions where resources are limited, and expensive scientific equipment is often not available. ColorX can democratise access to scientific knowledge and enable common people to conduct colorimetric experiments that were previously not possible. Furthermore, ColorX’s ultraportable form makes it easily transportable and eliminates the need for samples to be transported to a laboratory, which can be time-consuming and expensive. This can improve the speed and accuracy of test results, especially in environmental monitoring and process monitoring.

### 3.3. Limitations of ColorX

ColorX’s capability for colorimetric applications is currently restricted by the single green LED wavelength of the fitness tracker sensor used in this study. This spectral constraint limits analyte detection to colorimetric reagents with absorption bands that match the green LED emission spectrum, reducing the range of detectable analytes compared to benchtop spectrophotometers that access the full visible spectrum. Additionally, the device requires calibration for each new analyte type, and interference from other coloured species in complex samples has not been evaluated. In real-world samples, these interferences can affect the sensitivity. Additionally, the transreflectance geometry, while enabling compact design, inherently produces non-linear responses that may complicate quantitative analysis in some applications.

## 4. Conclusions

We have described ColorX, a low-cost and ultra-portable colorimetric sensing device built using a commercially available fitness tracker. By leveraging the built-in optoelectronic components of a fitness tracker, ColorX enables quantitative colorimetric analysis with high correlation to benchtop spectrophotometers (R^2^ values > 0.9 in all cases). Coefficients of variation ranged from 0.39 to 1.21 across all tested analytes, demonstrating excellent measurement precision. The system combines open-source firmware, a custom 3D-printed enclosure, and a Bluetooth-enabled Android app for wireless control, data acquisition, and visualization. The device achieved impressive analytical capabilities, with limits of detection (LODs) well within regulatory guidelines for key water quality indicators: 0.07 mg/L for nitrite (VIS PD), 18 mg/L for sulphate (IR PD), 0.005 mg/L for free chlorine (IR PD), 0.002 mg/L for chromium (IR PD), and 2.97 NTU for turbidity (VIS PD). Despite limitations in spectral flexibility and response linearity due to the transreflectance geometry, the device’s low cost, reusability, and open-source architecture make it a compelling platform for rapid, on-site colorimetric sensing. Overall, ColorX illustrates how consumer electronics can be reimagined as scientific tools, contributing to the democratisation of analytical instrumentation beyond traditional lab settings.

## Figures and Tables

**Table 1:** Calibrating ColorX: Calibration curves and corresponding R^2^ values for the five analytes tested with ColorX. The device demonstrated high calibration accuracy across all analytes, as evidenced by the strong R^2^ values obtained in the experiments.

Analyte	Calibration Equation	R^2^
**Nitrite**	[VIS] Absorbance = 1.631 + 0.07093[X] − 0.0003834[X]^2^	0.938
	[IR] Absorbance = 1.764 + 0.07325[X] − 0.0004441[X]^2^	0.905
	[Benchtop spec] Absorbance = 0.1082 + 0.03383[X] − 0.0001633[X]^2^	0.980
**Sulfate**	[VIS] Absorbance = 1.009 + 0.005449[X] − 8.708 × 10^−6^[X]^2^	0.996
	[IR] Absorbance = 0.995 + 0.004994[X] − 2.314 × 10^−6^[X]^2^	0.985
	[Benchtop spec] Absorbance = 0.005613[X] − 0.008083	0.918
**Free-Chlorine**	[VIS] Absorbance = 1.019 + 0.7936[X] − 0.07866[X]^2^	0.985
	[IR] Absorbance = 1.015 + 0.9859[X] − 0.1167[X]^2^	0.995
	[Benchtop spec] Absorbance = 0.2422[X] − 0.008548	0.997
**Chromium**	[VIS] Absorbance = 1.015 + 1.678[X] + 0.02142[X]^2^	0.900
	[IR] Absorbance = 1.014 + 0.8624[X] − 22.41[X]^2^	0.958
	[Benchtop spec] Absorbance = 0.8556[X] + 0.009704	0.959
**Turbidity**	[VIS]Absorbance = 1.031 + 1.173[X] − 0.582[X]^2^	0.990
	[IR]Absorbance = 1.031 + 1.121[X] − 0.5966[X]^2^	0.988
	[Benchtop spec] Absorbance = 1.329[X] + 0.02493	0.997

**Table 2:** Limits of detection (LOD) for various analytes using the ColorX device compared to EPA/CDC Guidelines. This table compares the EPA/CDC guidelines for the concentration of different analytes (nitrite, sulphate, free chlorine, chromium, and turbidity) in water within the limits of detection (LODs) using the ColorX device under a visible photodiode (Vis PD) and infrared photodiode (IR PD) conditions. (The LOD is calculated as the mean of blank values plus 3.3 times the standard deviation of the blank values divided by the slope of the calibration curve.) NTU values for the VIS and IR PD are interpolated from the standard curve obtained from known NTU ranges measured using a standard benchtop spectrophotometer. The %avgCV values for both VIS and IR PDs were less than 2 across all analytes, indicating an excellent precision of the ColorX device.

Analyte	EPA/CDC Guidelines	ColorX LOD and %Avg CV	
		VIS PD	IR PD
**NITRITE**	1 mg/L	0.07 mg/L, 1.06	0.1081 mg/L, 1.10
**SULFATE**	250 mg/L	46 mg/L, 0.39	18 mg/L, 0.75
**FREE CHLORINE**	44 mg/L	0.07 mg/L, 1.03	0.005 mg/L, 0.68
**CHROMIUM**	0.1 mg/L	0.018 mg/L, 0.69	0.002 mg/L, 051
**TURBIDITY**	1 NTU	2.97 NTU, 1.21	9.09 NTU, 1.04

## Data Availability

The original contributions presented in this study are included in the article. Further inquiries can be directed to the corresponding author. All the 3D, firmware, and application files for ColorX are released as open source under GPL 3.0 license and can be found here: https://github.com/V77VV/ColorX (accessed on 1 May 2024).
